# Lifestyle Factors and Associations with Individual and Comorbid Cardiometabolic and Pulmonary Disease Among U.S. Adults

**DOI:** 10.3390/ijerph21121674

**Published:** 2024-12-16

**Authors:** Osayande Agbonlahor, Delvon T. Mattingly, Maggie K. Richardson, Joy L. Hart, Alison C. McLeish, Kandi L. Walker

**Affiliations:** 1 Department of Preventive Medicine, University of Mississippi Medical Center, Jackson, MS 39216, USA; oagbonlahor@umc.edu; 2 Department of Behavioral Science, College of Medicine, University of Kentucky, Lexington, KY 40536, USA; delvonmattingly@uky.edu; 3 Center for Health, Engagement, and Transformation, College of Medicine, University of Kentucky, Lexington, KY 40536, USA; 4 Department of Educational, School, and Counseling Psychology, College of Education, University of Kentucky, Lexington, KY 40506, USA; maggie.richardson@uky.edu; 5 Department of Communication, College of Arts and Sciences, University of Louisville, Louisville, KY 40292, USA; kandi.walker@louisville.edu; 6 Christina Lee Brown Envirome Institute, School of Medicine, University of Louisville, Louisville, KY 40202, USA; 7 American Heart Association Tobacco Center for Regulatory Science, Dallas, TX 75231, USA; 8 Department of Psychological and Brain Sciences, University of Louisville, Louisville, KY 40292, USA; alison.mcleish@louisville.edu

**Keywords:** cardiometabolic disease, pulmonary disease, lifestyle factors, adults, sociodemographic characteristics

## Abstract

Background: Although lifestyle factors have been linked to chronic diseases among adults, their association with diagnosed individual and comorbid cardiometabolic (CMD) and pulmonary disease (PD) is not fully known. This study aimed to examine the associations between lifestyle factors and individual and comorbid CMD and PD among U.S. adults. Methods: We used cross-sectional data from the 2017–2020 National Health and Nutrition Examination Survey (*n* = 7394). Health care provider’s diagnosis of CMD and PD and lifestyle factors (i.e., past 5-day tobacco use, past 12-month alcohol use, diet, sleep troubles, and physical activity) were assessed. Adjusted odds ratios were estimated using logistic and multinomial logistic regression. Results: Trouble sleeping was associated with increased odds of CMD (OR: 2.47) and PD (OR: 2.29) individually, while physical activity was associated with lower odds (OR: 0.75, OR: 0.77). Past 5-day tobacco (OR: 2.36) and past year alcohol (OR: 1.61) use were associated with increased PD odds. Lifestyle factors were associated with increased odds of comorbid CMD and PD. Conclusions: Lifestyle factors were associated with increased odds of individual and comorbid CMD and PD among adults. CMD and PD prevention should involve promoting lifestyle modification and implementation of policies that eliminate structural barriers to healthy lifestyle adoption.

## 1. Introduction

Cardiometabolic disease (CMD) and pulmonary disease (PD) are the leading causes of mortality among adults in the United States (U.S.) [1,2,3]. CMD, which includes conditions such as coronary heart disease, stroke, hypertension, diabetes, obesity, and hypercholesterolemia, alters cardiovascular and metabolic functions [1]. PD, including chronic obstructive pulmonary disease (COPD), emphysema, chronic bronchitis, and asthma, is inflammatory in nature. Asthma onset primarily occurs in youth and is triggered by genetic factors, whereas COPD typically begins in adulthood and is influenced by environmental and lifestyle factors [4,5]. In the U.S., CMD is prevalent and affects approximately 50 million adults [6], whereas PD impacts 14.2 million adults [7]. According to recent estimates, annual direct and indirect U.S. healthcare costs are expected to exceed USD 422 billion for CMD [3] and USD 50 billion for PD [8]. Despite therapeutic efforts to control and mitigate the deleterious effects of these chronic diseases, comorbidity of CMD and PD persists among adults, posing an ever-increasing public health and socioeconomic burden [9,10]. As such, research and public health efforts have increasingly focused on prevention strategies, emphasizing lifestyle improvements over pharmaceutical and surgical interventions to address the detrimental impact of CMD and PD in the U.S. [2,11]. 

Indeed, lifestyle factors, such as physical inactivity, poor sleep, and substance use, have been identified as modifiable risk factors for CMD and PD [3,9,12,13]. Despite significant efforts to raise awareness about the importance of these factors in preventing chronic diseases, most U.S. adults still do not practice recommended healthy lifestyle behaviors [14,15]. Notably, 44.6% of adults are physically inactive [16], 33.8% report poor diet quality [17], 50.8% consumed alcohol in the past month [18], 18.7% currently use tobacco products, and 29.8% report trouble sleeping [3,19].

In addition, substantial disparities in lifestyle risk factors, CMD, and PD prevalence exist among sociodemographic population groups in the U.S. [7,15]. For example, CMD prevalence is higher among non-Hispanic Blacks, men, marginalized sexual or gender identities, and individuals with lower income [3]; PD prevalence is higher among women, individuals with lower education levels, and those who identify as non-Hispanic American Indian or Alaska Native [7]. Inequities in CMD and PD prevalence are typically borne by structurally marginalized populations predisposed to environmental and social conditions that perpetuate worse rates of physical inactivity, poor diet, substance use, and poor sleep [1,7].

Although previous research suggests that unhealthy lifestyle behavior may negatively impact cardiometabolic and pulmonary health, few studies have specifically examined the association between lifestyle factors and individual or comorbid CMD and PD among a nationally representative sample of U.S. adults. Thus, a comprehensive investigation of this association is warranted. Therefore, the current study aims to investigate the (1) associations between lifestyle factors and CMD and PD individually, (2) associations between lifestyle factors and comorbid CMD and PD, and (3) differences in socioenvironmental characteristics related to CMD and PD.

## 2. Materials and Methods

Data were obtained from the 2017–2020 National Health and Nutrition Examination Survey (NHANES), a cross-sectional, nationally representative survey of the noninstitutionalized U.S. civilian population. NHANES employed a complex, multistage, stratified cluster sample design to estimate the prevalence of health, nutritional status, and potential risk factors [20]. Data on sociodemographic characteristics, lifestyle characteristics, body measurements, and medical conditions were obtained from conducting household interviews and clinical examinations at a mobile examination center. NHANES was approved by the National Center for Health Statistics (NCHS) Research Ethics Review Board, and written informed consent was obtained from all participants before the interviews and clinical examinations. The current study was exempt from review by the University of Louisville Institutional Review Board as the data are deidentified and publicly available. 

### 2.1. Participants

**Figure 1:**
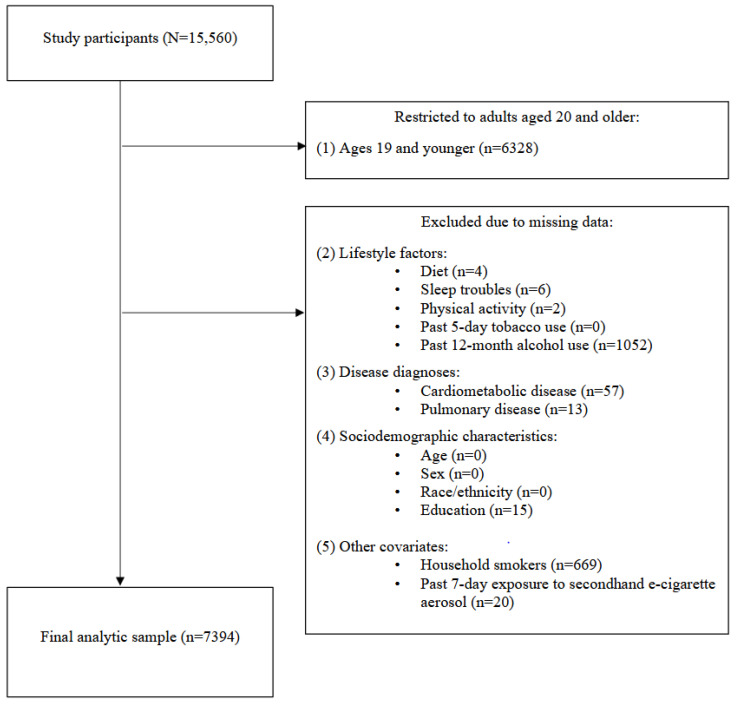
Flow chart of study.

A total of 15,560 participants (age range: 0–80 years, mean: 33.7 years; sex: 50.4% female, 49.6% male) completed the 2017–2020 NHANES. Because questions about CMD were administered only to those 20 years and older, we restricted the analytic sample to participants ages 20 and older (*n* = 9232). Participants were then excluded if they had missing information on socioenvironmental characteristics, lifestyle factors, and disease outcomes (*n* = 1838; missing data 19.9%). Details on exclusion criteria are shown in the study flow chart (see Figure 1). The final analytic sample included 7394 adults.

### 2.2. Primary Measures

#### 2.2.1. Cardiometabolic and Pulmonary Disease

CMD was defined as a self-reported healthcare provider diagnosis of one or more cardiovascular diseases (CVDs) (i.e., hypertension, coronary heart disease, stroke) or metabolic disease (i.e., diabetes, obesity, hypercholesterolemia). CVD diagnosis was obtained from participants’ responses (yes or no) to each of the following questions: “Have you ever been told by a doctor or other health professional that you had hypertension, also called high blood pressure?”, “Has a doctor or other health professional ever told you that you had coronary heart disease?”, and “Has a doctor or other health professional ever told you that you had stroke?”. Similarly, metabolic disease diagnosis was obtained from participants’ responses (yes or no) to each of the following questions: “Have you ever been told by a doctor or health professional that you have diabetes or sugar diabetes?”, “Has a doctor or other health professional ever told you that you were overweight?”, and “Have you ever been told by a doctor or other health professional that your blood cholesterol level was high?”. Participants who responded borderline to the diabetes question were included in the yes category, as prior research has shown pre-diabetes to be linked to lifestyle factors [21]. Therefore, we categorized CMD in the analysis as CMD (i.e., yes in response to one or more of the above questions) and no CMD (i.e., no in response to all six questions).

PD was defined from participants’ responses (yes or no) to the following question: “Have you ever been told by a doctor or other health professional that you had chronic obstructive pulmonary disease or COPD, emphysema, or chronic bronchitis?”. 

#### 2.2.2. Lifestyle Factors

Lifestyle factors included past 5-day tobacco use, past 12-month alcohol use, physical activity, diet, and sleep troubles. Participants were asked if they had used any tobacco product in the last five days, and those who responded yes identified which tobacco product(s) they used. We categorized past 5-day tobacco use as yes or no, which included the use of one or more of the following: cigarettes, electronic cigarettes (e-cigarettes), cigars, pipes, chewing tobacco, snuff, hookah or water pipes, other smokeless products, and nicotine patch or gum.

Past 12-month alcohol use was defined based on responses to the following questions: “In your entire life have you had at least one drink (i.e., 12 oz. beer, 5 oz. wine, or 1.5 ozs. of liquor) of any kind of alcohol, not counting small tastes or sips?”, and “During the past 12 months, about how often did you drink any type of alcoholic beverage?”. We categorized alcohol use as never, former use (i.e., use in lifetime but not during the past 12 months), and past year use (i.e., used at least once in the past 12 months).

Physical activity was defined based on participants’ responses (yes or no) to each of the following questions: “In a typical week do you do any moderate-intensity sports, fitness, or recreational activities that cause a small increase in breathing or heart rate such as brisk walking, bicycling, swimming or volleyball for at least 10 min continuously?” and “In a typical week do you do any vigorous-intensity sports, fitness, or recreational activities that cause large increases in breathing or heart rate like running or basketball for at least 10 min continuously?”. We categorized physical activity in the analysis as yes (i.e., yes in response to one or both questions) and no (i.e., no to both questions).

Diet was defined based on participants’ responses (excellent, very good, good, fair, or poor) to the following question: “In general, how healthy is your overall diet?”. We categorized diet as *good* (i.e., good, very good, or excellent) and *poor* (i.e., fair or poor). In addition, we defined sleep troubles based on responses (yes or no) to the question “Have you ever told a doctor or other health professional that you have trouble sleeping?”.

#### 2.2.3. Sociodemographic and Environmental Characteristics

Sociodemographic characteristics included age (20–34 years, 35–49 years, 50–59 years, 60 years and older), sex (female, male), education level completed (<high school, high school graduate/GED, associate degree, college graduate), and race/ethnicity (Mexican American, Other Hispanic, Non-Hispanic White, Non-Hispanic Black, Other races/ethnicities). Other races/ethnicities included Non-Hispanic Asian, Non-Hispanic multi-racial, and those not self-identifying as any of the above-mentioned races/ethnicities. Environmental characteristics assessed were the presence of household smokers (yes or no) and past seven-day exposure to secondhand e-cigarette aerosol (yes or no).

### 2.3. Statistical Analysis

Weighted percentages and confidence intervals of lifestyle factors, CMD and PD diagnoses, and socioenvironmental characteristics were examined. An analysis of socioenvironmental characteristics by each disease diagnosis was performed using Chi-square tests of independence or Fisher’s exact tests when appropriate. Multivariable logistic regression models were performed to examine associations between lifestyle factors and each chronic disease (CMD and PD); the outcome referent group for each model was no disease. Two multinomial logistic regression models were used to investigate the association between lifestyle factors and comorbid CMD and PD; the outcome referent group was no disease (model one) and individual CMD or PD (model two). Both sets of models were adjusted for age, sex, race and ethnicity, educational level completed, household smokers, and past seven-day exposure to secondhand e-cigarette aerosol. Adjusted odds ratios and 95% confidence intervals are reported. Results were considered statistically significant at α = 0.05. Data were analyzed using Stata 17.0. NHANES complex survey design was accounted for, and the analyses were adjusted for the probability of non-response by using the *svy* command. As recommended by NHANES analytic guidelines [22], the sample weights specific to the NHANES 2017–March 2020 pre-pandemic data were used.

## 3. Results

### 3.1. Descriptive Characteristics

Distributions of socioenvironmental characteristics, lifestyle factors, and disease outcomes are displayed in Table 1. Among the analytic sample of U.S. adults, most were aged 60 and older (29.7%), female (51.6%), Non-Hispanic White (64.3%), college graduates (32.1%), not residing in households with smokers (71.0%), not exposed to secondhand e-cigarette aerosol in the past seven days (84.5%), and never tobacco users (77.4%). Of the participants who reported using tobacco (22.6%) in the past five days, 14.3% used cigarettes, 3.2% used e-cigarettes, 3.0% used other tobacco products, and 2.1% used cigars. Furthermore, 55.3% of adults in the sample reported physical inactivity, 30.8% reported trouble sleeping, 30.6% reported poor diet, and 77.1% reported alcohol use in the past 12 months. Regarding disease outcomes, 58.3% reported a diagnosis of CMD, 8.7% a diagnosis of PD, and 6.9% comorbid CMD and PD.

Results for each of the diseases differed by age, race/ethnicity, and education completed. For example, more adults ages 60 years and older than adults ages 20–34 (*p* ≤ 0.001) and identifying as Non-Hispanic Black than Mexican American (*p* = 0.038) had CMD and more adults who completed high school than college (*p* = 0.045) and identified as Non-Hispanic White than Mexican American (*p* ≤ 0.001) had PD (see Supplemental Table S1).

### 3.2. Lifestyle Factors and Cardiometabolic Disease

Results of the multivariable logistic regression estimating associations between lifestyle factors and CMD are shown in Table 2. After adjusting for socioenvironmental characteristics, adults who had trouble sleeping (OR: 2.47, 95% CI: 2.17–2.82), poor diet (OR: 1.80, 95% CI: 1.48–2.18), and formerly used alcohol (OR: 1.67, 95% CI: 1.25–2.24) had higher odds of CMD compared with those with no trouble sleeping, good diet, and never alcohol use, respectively. Adults who were physically active had lower odds of CMD (OR: 0.75, 95% CI: 0.62–0.91) compared with adults who were physically inactive.

Adults ages 20–34 years (OR: 0.22, 95% CI: 0.17–0.28), 35–49 years (OR: 0.37, 95% CI: 0.29–0.48), and 50–59 years (OR: 0.56, 95% CI: 0.39–0.80) had lower odds of CMD compared with adults 60 years and older. Compared with Non-Hispanic White adults, Non-Hispanic Black adults had higher odds of CMD (OR: 1.29, 95% CI: 1.02–1.62). Compared with adults with less than high school education completed, adults with high school/GED completed (OR: 1.37, 95% CI: 1.06–1.77) and adults with an associate degree (OR: 1.58, 95% CI: 1.26–1.99) had higher odds of CMD. 

### 3.3. Lifestyle Factors and Pulmonary Disease

Results of the multivariable logistic regression estimating associations between lifestyle factors and PD are shown in Table 3. After adjusting for socioenvironmental characteristics, adults who used tobacco in the past 5 days (OR: 2.36, 95% CI: 1.58–3.53), formerly used alcohol (OR: 2.90, 95% CI: 1.84–4.59), or used alcohol in the past year (OR: 1.61, 95% CI: 1.06–2.43) had higher odds of PD compared with those who did not use any tobacco product in the past 5 days and never used alcohol. In addition, adults who had trouble sleeping (OR: 2.29, 95% CI: 1.86–2.82) had higher odds of PD compared with those with no trouble sleeping, and adults who were physically active (OR: 0.77, 95% CI: 0.62–0.96) had lower odds of PD compared with adults who were physically inactive.

Adults ages 20–34 years (OR: 0.15, 95% CI: 0.08–0.27), 35–49 years (OR: 0.25, 95% CI: 0.16–0.38), and 50–59 years (OR: 0.40, 95% CI: 0.26–0.62) had lower odds of PD compared with adults 60 years and older, and Mexican American adults (OR: 0.35, 95% CI: 0.21–0.57) had lower odds of PD compared with Non-Hispanic White adults. Further, adults who were college graduates had lower odds of PD (OR: 0.51, 95% CI: 0.31–0.83), and adults who lived in a household where members smoke had higher odds of PD (OR: 1.57, 95% CI: 1.13–2.19). 

### 3.4. Lifestyle Factors and Comorbid CMD and Pulmonary Disease

Results of the multinomial logistic regression estimating associations between lifestyle factors and CMD and PD comorbidity are shown in Table 4. After adjustment for socioenvironmental characteristics, adults who had trouble sleeping (OR: 5.12, 95% CI: 4.10–6.38), reported poor diet (OR: 1.98, 95% CI: 1.43–2.75), used tobacco in the past 5 days (OR: 1.78, 95% CI: 1.09–2.90), formerly used alcohol (OR: 4.52, 95% CI: 2.77–7.39), and used alcohol in the past year (OR: 1.93, 95% CI: 1.15–3.25) had higher odds of comorbid CMD and PD (vs. no disease) compared with those who had no trouble sleeping, good diet, did not use any tobacco product in the past 5 days, and never used alcohol. In addition, adults who were physically active (OR: 0.59, 95% CI: 0.43–0.80) had lower odds of CMD and PD disease comorbidity (vs. no disease) compared with adults who were physically inactive. 

Further, adults who had trouble sleeping (OR: 2.13, 95% CI: 1.66–2.74), used tobacco in the past 5 days (OR: 2.03, 95% CI: 1.34–3.07), and formerly used alcohol (OR: 2.80, 95% CI: 1.88–4.18) had higher odds of comorbid CMD and PD (vs. individual CMD or PD) compared with those who had no trouble sleeping, did not use any tobacco product in the past 5 days, and never used alcohol. 

## 4. Discussion

We examined the association between lifestyle factors, CMD, and PD among a nationally representative sample of U.S. adults, adjusting for socioenvironmental characteristics. We found that a significant number of U.S. adults engage in unhealthy behaviors (e.g., 22.6% used tobacco in the past 5 days), which is a cause for concern due to the impact that unhealthy lifestyle behaviors may have on cardiometabolic [14,23] and pulmonary [24,25] health outcomes. Indeed, our study’s findings indicate that trouble sleeping, poor diet, physical inactivity, and lifetime alcohol use were linked to a diagnosis of CMD, and past 5-day tobacco use, lifetime and past year alcohol use, trouble sleeping, and physical inactivity were linked to a diagnosis of PD. Further, trouble sleeping, poor diet, substance use, and physical inactivity were linked to comorbid CMD and PD. Notably, sleep troubles and substance use were also associated with comorbid CMD and PD as compared to diagnosis of an individual disease. Overall, these findings suggest that lifestyle behaviors persist as factors related to individual and comorbid CMD and PD among U.S. adults; thus, a comprehensive understanding of and well-honed approaches for addressing such factors are critical for the prevention and management of these chronic diseases.

Sleep is increasingly recognized as an essential lifestyle determinant of health outcomes [14], and our finding of higher odds of comorbid CMD and PD among adults with trouble sleeping provides further evidence supporting this idea. Previous research has documented that trouble sleeping is associated with CMD in isolation [26], but no study, to our knowledge, has examined its links to CMD and PD comorbidity. Pathophysiological mechanisms potentially underlying the association between poor sleep and comorbid CMD and PD include disruption of circadian rhythms resulting in hypoxia-induced activation of proinflammatory pathways, adipose tissue lipolysis, increased sympathetic drive, elevated cortisol concentrations, endothelial dysfunction, and increased oxidative stress [9,27,28]. Additional research is needed to better understand the mechanisms behind the association between trouble sleeping and comorbid CMD and PD. Further, our findings align with related work that found physical inactivity was associated with PD independently among adults in China [29] and that poor diet and substance use were associated with CMD independently among adults in sub-Saharan Africa [30]. Taken together, these studies suggest that reducing sedentary time, improving diet quality, and ceasing or lessening substance use are important regardless of location and cost-effective approaches for improving pulmonary and cardiometabolic health. 

Similar to some previous research, our study found CMD disparities for non-Hispanic Black adults compared to non-Hispanic White adults [1,15,31,32]. Such inequities can be traced to structural and systemic causes [33,34], with low rates of educational attainment, neighborhood disadvantage, and economic instability shaping lifestyle options available to racial or ethnic minorities (e.g., access to fully-stocked grocery stores and health care) compared to options available to their White counterparts [34]. Further, non-Hispanic Black adults are less likely to have access to safe areas for walking and other physical activity and more likely to have sleep disturbances (due to racial trauma), experience food insecurity, and have tobacco and liquor stores strategically placed in their neighborhoods [34,35,36]. Therefore, culturally sensitive policies that aim to address and eliminate structural barriers to healthy lifestyle behaviors are important for reducing the chronic disease disparities unequally borne by racial and ethnic marginalized populations. Additionally, until systemic change is achieved, public health efforts promoting lifestyle modifications in racial/ethnic populations are important in CMD and PD management and prevention [37,38].

Notably, the chronic disease disparities observed for individuals with low educational levels and in older adults are analogous to previous research [3,7]. Although prior studies have found associations between environmental determinants, such as living in a household with smokers and exposure to secondhand vaping, and CMD and PD [39,40], our study did not find evidence other than an association between household smokers and PD that corroborates those results. Future research that employs longitudinal and prospective cohort designs may be useful in further exploring sociodemographic and environmental determinants of CMD and PD development among U.S. adults. 

Implementing equitable policies and approaches that consider the social and environmental disadvantages that perpetuate unhealthy lifestyle behaviors among minority populations is critical for addressing the significant burden of CMD and PD. Community-based interventions are needed to increase the availability of and access to healthy foods and beverages, provide safe and convenient places for physical activity and sleep, and limit the presence of alcohol and tobacco stores in neighborhoods predominantly occupied by marginalized and underserved populations. When systemic barriers to healthy behavior are removed, positive lifestyle behaviors are likely to increase and thus reduce CMD disparities [1,34]. Further, utilizing lifestyle modifications as a preventive or ameliorative strategy for addressing CMD and PD is critical [11,15,24], as is enlisting health care practitioners to provide equitable lifestyle modification advice to all adults and youth. Interventions that improve the accessibility and quality of healthcare provider counseling and health promotion campaigns encouraging healthy lifestyle behaviors may also ensure that more adults know the impact that lifestyle factors have on preventing disease and improving health outcomes and overall quality of life. Finally, more emphasis in research and practice should be placed on the importance of sleep as a key lifestyle behavior for improving cardiometabolic and pulmonary health among adults. 

### Limitations

The results of this study should be interpreted considering the following limitations. First, the NHANES data are cross-sectional; thus, causality cannot be determined. Second, the study relied on self-reported responses on lifestyle factors, CMD, PD, and socioenvironmental characteristics, which may be subject to recall and response bias. Third, the study’s inclusion/exclusion criteria may have limited generalizability. 

## 5. Conclusions

This study found that lifestyle factors are associated with individual and comorbid CMD and PD diagnosis. Further, adults with trouble sleeping and those who used substances such as tobacco and alcohol were more likely to have comorbid CMD and PD. Considering these findings, the development and implementation of comprehensive policies and interventions focusing on eliminating structural and individual barriers to healthy lifestyle behaviors and promoting lifestyle modification as a cost-effective prevention and management strategy for CMD and PD are needed. Further, public health efforts should focus on highlighting sleep as a key lifestyle behavior for improving adult cardiometabolic and pulmonary health.

## Figures and Tables

**Table 1:** Distributions of participant characteristics, lifestyle factors, and cardiometabolic and pulmonary disease among adults in the United States, NHANES 2017–2020 (*n* = 7394).

Variables	*n* (%)	95% CI
Age (years)		
20–34	1712 (27.4)	25.2, 29.6
35–49	1715 (24.7)	22.6, 27.0
50–59	1278 (18.2)	16.5, 19.9
60 and older	2689 (29.7)	26.6, 33.2
Sex		
Male	3617 (48.4)	46.7, 50.1
Female	3777 (51.6)	49.9, 53.3
Education level completed		
<High school	1309 (10.3)	9.2, 11.4
High school/GED	1799 (27.1)	24.3, 30.2
Associate degree	2458 (30.5)	28.6, 32.6
College degree	1828 (32.1)	27.7, 36.8
Race/ethnicity		
Mexican American	864 (8.2)	6.1, 11.0
Other Hispanic	750 (7.3)	5.9, 8.9
NH White	2656 (64.3)	59.3, 69.0
NH Black	1951 (10.9)	8.4, 14.2
Other races or ethnicities	1173 (9.3)	7.5, 11.4
CMD		
No	2784 (41.7)	39.3, 44.2
Yes	4610 (58.3)	55.8, 60.7
PD		
No	6712 (91.3)	90.1, 92.4
Yes	682 (8.7)	7.6, 9.9
Chronic disease morbidity		
No disease	2653 (39.9)	37.4, 42.5
Comorbid CMD and PD	551 (6.9)	5.9, 8.0
Individual CMD or PD	4190 (53.2)	50.9, 55.5
Past 5-day tobacco use		
No	5646 (77.4)	75.0, 79.7
Yes	1748 (22.6)	20.3, 25.0
Past 12-month alcohol use		
Never	675 (6.7)	5.9, 7.7
Former	1486 (16.2)	14.9, 17.5
Past year	5233 (77.1)	75.7, 78.5
Diet		
Good	4927 (69.4)	67.4, 71.3
Poor	2467 (30.6)	28.7, 32.6
Sleep troubles		
No	5223 (69.2)	67.0, 71.2
Yes	2171 (30.8)	28.8, 33.0
Physical activity		
No	3876 (55.3)	52.4, 58.0
Yes	3518 (44.7)	42.0, 47.6
Household smokers		
No	5045 (71.0)	67.6, 74.3
Yes	2349 (29.0)	25.7, 32.4
Past 7-day secondhand e-cigarette exposure		
No	6386 (84.5)	82.1, 86.7
Yes	1008 (15.5)	13.3, 17.9

Weighted percentages and confidence intervals; unweighted frequencies; NHANES = National Health and Nutrition Examination Survey; NH = Non-Hispanic; other races/ethnicities = NH Asian, NH Multi-racial, any other race or ethnicity other than those mentioned above; CMD = cardiometabolic disease (i.e., health care provider diagnosis of one or more: stroke, high blood pressure, coronary heart disease, diabetes, obesity, or high cholesterol); PD = pulmonary disease (i.e., health care provider diagnosis of chronic obstructive pulmonary disease, emphysema or chronic bronchitis); past 5-day tobacco use = past 5-day use of any of the following tobacco products: cigarettes, electronic cigarettes, cigars, pipes, chewing tobacco, snuff, hookah or water pipes, other smokeless products, nicotine patch or gum; past 12-month alcohol use = past 12-month use of any kind of alcohol; diet = general quality of overall diet; physical activity = performing moderate-intensity and/or vigorous-intensity activities in a typical week; past 7-day secondhand e-cigarette exposure = past 7-day exposure to indoor electronic vaping product use.

**Table 2:** Multivariable logistic regression estimating associations between lifestyle factors and cardiometabolic disease (*n* = 7394).

	Cardiometabolic Disease (CMD) ^a^	
	OR ^b^	95% CI
Past 5-day tobacco use (ref: no)		
Yes	0.83	0.66, 1.04
Past 12-month alcohol use (ref: never)		
Former alcohol use	**1.67**	**1.25, 2.24**
Past year alcohol use	1.27	0.97, 1.66
Age (ref: 60 years and older)		
20–34 years	**0.22**	**0.17, 0.28**
35–49 years	**0.37**	**0.29, 0.48**
50–59 years	**0.56**	**0.39, 0.80**
Sex (ref: male)		
Female	0.96	0.80, 1.15
Race/ethnicity (ref: non-Hispanic White)		
Mexican American	1.18	0.91, 1.53
Other Hispanic	0.94	0.77, 1.15
Non-Hispanic Black	**1.29**	**1.02, 1.62**
Other races/ethnicities ^c^	1.11	0.91, 1.35
Education completed (ref: less than high school)		
High school/GED	**1.37**	**1.06, 1.77**
Associate degree	**1.58**	**1.26, 1.99**
College degree	1.12	0.87, 1.44
Sleep troubles (ref: no)		
Yes	**2.47**	**2.17, 2.82**
Physical activity (ref: no)		
Yes	**0.75**	**0.62, 0.91**
Diet (ref: good)		
Poor	**1.80**	**1.48, 2.18**
Household smokers (ref: no)		
Yes	0.90	0.69, 1.18
Past 7-day secondhand e-cigarette exposure (ref: no)		
Yes	1.12	0.81, 1.55

^a^ Outcome referent group: no CMD (i.e., stroke, hypertension, coronary heart disease, diabetes, obesity, hypercholesterolemia). ^b^ Model is adjusted for age, sex, educational level, race/ethnicity, household smoking, and secondhand e-cigarette exposure. ^c^ Other races/ethnicities include respondents who identified as NH Asian, NH Multi-racial, or any other race or ethnicity other than those mentioned above. Bolded numbers indicate statistical significance (i.e., *p*-value of <0.05).

**Table 3:** Multivariable logistic regression estimating associations between lifestyle factors and pulmonary disease (*n* = 7394).

	Pulmonary Disease (PD) ^a^	
	OR ^b^	95% CI
Past 5-day tobacco use (ref: no)		
Yes	**2.36**	**1.58, 3.53**
Past 12-month alcohol use (ref: never)		
Former alcohol use	**2.90**	**1.84, 4.59**
Past year alcohol use	**1.61**	**1.06, 2.43**
Age (ref: 60 years and older)		
20–34 years	**0.15**	**0.08, 0.27**
35–49 years	**0.25**	**0.16, 0.38**
50–59 years	**0.40**	**0.26, 0.62**
Sex (ref: male)		
Female	1.20	0.83, 1.73
Race/ethnicity (ref: non-Hispanic White)		
Mexican American	**0.35**	**0.21, 0.57**
Other Hispanic	**0.70**	**0.50, 0.99**
Non-Hispanic Black	0.69	0.48, 1.01
Other races/ethnicities ^c^	1.00	0.76, 1.32
Education completed (ref: less than high school)		
High school/GED	1.23	0.92, 1.64
Associate degree	0.89	0.62, 1.28
College degree	**0.51**	**0.31, 0.83**
Sleep troubles (ref: no)		
Yes	**2.29**	**1.86, 2.82**
Physical activity (ref: no)		
Yes	**0.77**	**0.62, 0.96**
Diet (ref: good)		
Poor	1.20	0.94, 1.52
Household smokers (ref: no)		
Yes	**1.57**	**1.13, 2.19**
Past 7-day secondhand e-cigarette exposure (ref: no)		
Yes	1.41	0.89, 2.23

^a^ Outcome referent group: no PD (i.e., chronic obstructive pulmonary disease/COPD, emphysema, chronic bronchitis). ^b^ Model is adjusted for age, sex, educational level, race/ethnicity, household smoking, and secondhand e-cigarette exposure. ^c^ Other races/ethnicities include respondents who identified as NH Asian, NH Multi-racial, or any other race or ethnicity other than those mentioned above. Bolded numbers indicate statistical significance (i.e., *p*-value of <0.05).

**Table 4:** Multinomial logistic regression estimating associations between lifestyle factors and comorbidity of cardiometabolic and pulmonary disease (*n* = 7394).

	Comorbid CMD and PD ^a^		Individual CMD or PD ^a^		Comorbid vs. Individual CMD or PD ^b^	
	OR ^c^	95% CI	OR ^c^	95% CI	OR ^c^	95% CI
Past 5-day tobacco use (ref: no)						
Yes	**1.78**	**1.09, 2.90**	0.88	0.66, 1.16	**2.03**	**1.34, 3.07**
Past 12-month alcohol use (ref: never)						
Former alcohol use	**4.52**	**2.77, 7.39**	**1.61**	**1.20, 2.18**	**2.80**	**1.88, 4.18**
Past year alcohol use	**1.93**	**1.15, 3.25**	1.28	0.96, 1.70	1.51	0.97, 2.35
Age (ref: 60 years and older)						
20–34 years	**0.05**	**0.02, 0.10**	**0.22**	**0.17, 0.27**	**0.21**	**0.10, 0.48**
35–49 years	**0.13**	**0.08, 0.21**	**0.35**	**0.27, 0.46**	**0.37**	**0.23, 0.60**
50–59 years	**0.25**	**0.13, 0.46**	**0.56**	**0.39, 0.79**	**0.44**	**0.28, 0.70**
Sex (ref: male)						
Female	1.12	0.74, 1.71	0.97	0.81, 1.16	1.15	0.83, 1.60
Race/ethnicity (ref: non-Hispanic White)						
Mexican American	**0.44**	**0.24, 0.79**	1.18	0.91, 1.53	**0.37**	**0.22, 0.63**
Other Hispanic	**0.70**	**0.50, 0.98**	0.93	0.76, 1.14	0.75	0.52, 1.07
Non-Hispanic Black	0.94	0.57, 1.55	**1.25**	**1.01, 1.57**	0.75	0.52, 1.09
Other races/ethnicities ^d^	1.01	0.70, 1.47	1.15	0.92, 1.43	0.88	0.60, 1.30
Education completed (ref: <high school)						
High school/GED	**1.54**	**1.03, 2.30**	**1.42**	**1.07, 1.88**	1.09	0.77, 1.53
Associate degree	1.30	0.82, 2.07	**1.62**	**1.26, 2.07**	0.80	0.54, 1.19
College degree	0.54	0.28, 1.03	1.15	0.89, 1.50	**0.47**	**0.27, 0.82**
Sleep troubles (ref: no)						
Yes	**5.12**	**4.10, 6.38**	**2.40**	**2.09, 2.75**	**2.13**	**1.66, 2.74**
Physical activity (ref: no)						
Yes	**0.59**	**0.43, 0.80**	**0.77**	**0.62, 0.94**	0.77	0.57, 1.04
Diet (ref: good)						
Poor	**1.98**	**1.43, 2.75**	**1.80**	**1.46, 2.22**	1.10	0.87, 1.42
Household smokers (ref: no)						
Yes	1.42	0.86, 2.35	0.90	0.68, 1.18	**1.59**	**1.08, 2.33**
Past 7-day secondhand e-cigarette exposure (ref: no)						
Yes	1.56	0.83, 2.94	1.11	0.82, 1.49	1.41	0.86, 2.30

CMD = cardiometabolic disease; PD = pulmonary disease; comorbid CMD and PD = diagnosis of CMD and PD; individual CMD or PD = diagnosis of CMD only or PD only. ^a^ Outcome referent group: no disease (i.e., cardiometabolic disease, pulmonary disease). ^b^ Outcome referent group: individual CMD or PD (i.e., diagnosis of CMD only or PD only). ^c^ Model is adjusted for age, sex, educational level, race/ethnicity, household smoking, and secondhand e-cigarette exposure. ^d^ Other races/ethnicities include respondents who identified as NH Asian, NH Multi-racial, or any other race or ethnicity other than those mentioned above. Bolded numbers indicate statistical significance (i.e., *p*-value of <0.05).

## Data Availability

The data used in this study are available upon request from the corresponding author.

## Supplementary Materials

The following supporting information can be downloaded at https://www.mdpi.com/article/10.3390/ijerph21121674/s1, Table S1: Sociodemographic and Environmental Factors by Cardiometabolic and Pulmonary Disease, NHANES 2017-2020 (n = 7394).
