# Supplementary material for: Lifestyle Factors and Associations with Individual and Comorbid Cardiometabolic and Pulmonary Disease Among U.S. Adults
Source: Int J Environ Res Public Health. 2024 Dec 16;21(12):1674. doi: 10.3390/ijerph21121674 (PMC11675608; doi:10.3390/ijerph21121674)
Supplement: Supplementary file 1 [file ijerph-21-01674-s001.zip › ijerph-3359658-supplementary.pdf]

Supplemental Table S1: Sociodemographic and Environmental Factors by Cardiometabolic and Pulmonary Disease, NHANES 2017-2020 (n=7,394)

	**Cardiometabolic disease**			**Pulmonary Disease**		
**Variables**	**No (N=2784)**	**Yes (N=4610)**	**P Value**	**No (N=6712)**	**Yes (N=682)**	**P Value**
**Age (years)**			**< 0.001**			**<0.001**
20-34 (%)	1043 (39.5)	669 (18.7)		1661 (29.0)	51 (9.9)	
35-49 (%)	767 (27.6)	948 (22.6)		1624 (25.7)	91 (14.6)	
50-59 (%)	406 (15.5)	872 (20.1)		1152 (18.1)	126 (19.0)	
60+ (%)	568 (17.4)	2121 (38.6)		2275 (27.2)	414 (56.5)	
**Sex**			0.479			0.332
Male (%)	1417 (49.2)	2200 (47.8)		3283 (48.7)	334 (44.6)	
Female (%)	1367 (50.8)	2410 (52.2)		3429 (51.3)	348 (55.4)	
**Education completed**			**<0.001**			**0.045**
Less than high school (%)	511 (10.4)	798 (10.2)		1165 (10.0)	144 (12.9)	
High school (%)	659 (24.8)	1140 (28.8)		1584 (25.7)	215 (42.0)	
Associate degree (%)	835 (27.5)	1623 (32.7)		2212 (30.5)	246 (31.0)	
College degree (%)	779 (37.3)	1049 (28.4)		1751 (33.8)	77 (14.2)	
**Race/ethnicity**			**0.038**			**<0.001**
Mexican American (%)	343 (8.8)	521 (7.9)		838 (8.8)	26 (2.3)	
Other Hispanic (%)	304 (7.9)	446 (6.8)		698 (7.5)	52 (5.0)	
NH White (%)	931 (63.1)	1725 (65.1)		2278 (63.3)	378 (75.3)	
NH Black (%)	655 (10.1)	1296 (11.5)		1795 (11.1)	156 (9.0)	
Other races/ethnicities (%)	551 (10.1)	622 (8.7)		1103 (9.3)	70 (8.4)	
**Household smokers**			0.350			**<0.001**
No (%)	1853 (69.8)	3192 (71.9)		4686 (72.8)	359 (52.7)	
Yes (%)	931 (30.2)	1418 (28.1)		2026 (27.2)	323 (47.3)	
**Past 7-day secondhand e-cigarette exposure**			0.217			0.272
No (%)	2363 (83.3)	4023 (85.4)		5808 (84.8)	578 (81.6)	

	Cardiometabolic disease			Pulmonary Disease		
**Variables**	**No (N=2784)**	**Yes (N=4610)**	**P Value**	**No (N=6712)**	**Yes (N=682)**	**P Value**
Yes (%)	421 (16.7)	587 (14.6)		904 (15.2)	104 (18.4)	

Weighted percentages and confidence intervals; unweighted frequencies; NHANES= National Health and Nutrition Examination Survey; e-cigarette= electronic cigarette; NH= Non-Hispanic; Other races/ethnicities= NH Asian, NH Multi-racial, any other race or ethnicity other than those mentioned above; CMD = cardiometabolic disease (i.e., health care provider diagnosis of one or more: stroke, high blood pressure, coronary heart disease, diabetes, obesity, or high cholesterol); PD = pulmonary disease (i.e., health care provider diagnosis of chronic obstructive pulmonary disease, emphysema or chronic bronchitis); past 7-day secondhand e-cigarette exposure = past 7-day exposure to indoor electronic vaping product use
